# Fish biodiversity declines with dam development in the Lower Mekong Basin

**DOI:** 10.1038/s41598-023-35665-9

**Published:** 2023-05-26

**Authors:** Ratha Sor, Peng Bun Ngor, Sovan Lek, Kimsan Chann, Romduol Khoeun, Sudeep Chandra, Zeb S. Hogan, Sarah E. Null

**Affiliations:** 1 Department of Watershed Sciences, Utah State University, Logan, UT 84322 USA; 2 Graduate School, National University of Cheasim Kamchaymear, No. 157, Preah Norodom Blvd, Khan Chamkarmon, Phnom Penh, 12300 Cambodia; 3 Wonders of the Mekong Project, C/O IFReDI, Fisheries Administration, No. 186, Preah Norodom Blvd., Khan Chamkar Morn, Phnom Penh, 12300 Cambodia; 4 Faculty of Fisheries, Royal University of Agriculture, Sangkat Dongkor, Khan Dongkor, P.O. Box 2696, Phnom Penh, 120501 Cambodia; 5 Université de Toulouse, Laboratoire Evolution & Diversité Biologique, UMR 5174, CNRS-Université Paul Sabatier, 118 Route de Narbonne, 31062 Toulouse Cédex 4, France; 6 Faculty of Hydrology and Water Resources Engineering, Institute of Technology of Cambodia, Russian Federation Boulevard, Phnom Penh, 12156 Cambodia; 7 Global Water Center & Department of Biology, University of Nevada, 1664 N. Virginia Street, Reno, NV 89557 USA

**Keywords:** Freshwater ecology, Biodiversity

## Abstract

Hydropower dams are a source of renewable energy, but dam development and hydropower generation negatively affect freshwater ecosystems, biodiversity, and food security. We assess the effects of hydropower dam development on spatial–temporal changes in fish biodiversity from 2007 to 2014 in the Sekong, Sesan, and Srepok Basins—major tributaries to the Mekong River. By analyzing a 7-year fish monitoring dataset, and regressing fish abundance and biodiversity trends against cumulative number of upstream dams, we found that hydropower dams reduced fish biodiversity, including migratory, IUCN threatened and indicator species in the Sesan and Srepok Basins where most dams have been constructed. Meanwhile, fish biodiversity increased in the Sekong, the basin with the fewest dams. Fish fauna in the Sesan and Srepok Basins decreased from 60 and 29 species in 2007 to 42 and 25 species in 2014, respectively; while they increased from 33 in 2007 to 56 species in 2014 in the Sekong Basin. This is one of the first empirical studies to show reduced diversity following dam construction and fragmentation, and increased diversity in less regulated rivers in the Mekong River. Our results underscore the importance of the Sekong Basin to fish biodiversity and highlight the likely significance of all remaining free-flowing sections of the Lower Mekong Basin, including the Sekong, Cambodian Mekong, and Tonle Sap Rivers to migratory and threatened fish species. To preserve biodiversity, developing alternative renewable sources of energy or re-operating existing dams to increase power generation are recommended over constructing new hydropower dams.

## Introduction

The Mekong River (hereafter the Mekong) is one of the world’s most important biodiversity hotspots. It supports ~ 1200 fish species^1^, most of which live in the Lower Mekong Basin (LMB). The LMB is also home to a rich diversity of other aquatic fauna such as over 400 species of micro-invertebrates^2^, and at least 300 macroinvertebrate species such as molluscs^3^, crustaceans, annelids and aquatic insects^4–6^. In the LMB, fish are instrumental to the economy and provide food security for local people^7,8^. An estimated 1.3–2.7 million tonnes of fish are harvested from the LMB, valued at ~ $11 billion US dollars (USD) per year in 2015, or approximately $12.9 billion when adjusted for inflation in 2022^8^. However, fish biodiversity and fisheries productivity in the LMB are threatened by hydropower development. Dams disrupt and block fish migrations and alter fish communities and aquatic ecosystems, leading to decreased fish biodiversity, abundance, and biomass^9^. The decrease in fish yield is estimated to reduce biomass by 725,000 tonnes per year^10^, affecting food security, biodiversity, and ecosystem services in the Mekong^7,11^. This biomass reduction corresponds to an annual GDP loss of about $326 million USD in Cambodia and $165 million USD in Vietnam^12^.

Hydropower dam development projects are booming in the Mekong because increasing energy production is important for the region development. In the LMB, there are 129 commissioned dams, including 5 mainstem Mekong River dams in Laos. All told, 69 projects are located in Laos, 46 in Vietnam, 10 in Thailand, and 4 in Cambodia, which together have capacity to generate 13,340 megawatts (MW) of hydropower^13^. Moreover, 31 additional hydropower dams are under construction, including three on the mainstem Mekong River in Laos. Another 19 large dams (> 200 MW) have been proposed across the LMB. By 2040, estimated economic gain from all projects in the LMB will exceed $160 billion USD, while their potential cost is about $170 billion USD due to declining fisheries, forests, wetlands and mangroves^14^. Overall, a net economic loss is anticipated from hydropower projects, with the vast majority of benefits accruing to foreign companies and the majority of losses borne by residents of Cambodia, Thailand, and Vietnam, especially rural subsistence fishers who depend on fisheries for food, protein, and livelihoods^10^.

The impact of dams on the Mekong’s fish and fisheries have been investigated and summarized in several recent studies. Ziv et al.^7^ estimated fish biomass and biodiversity losses based on future dam alternatives and estimated trade-offs between power production and impacts to fish biodiversity. Kano et al.^15^ modeled the future distribution of fish with intense hydropower development and found reduced species richness and available habitat. Ngor et al.^9^ assessed the impacts of flow alteration on fish diversity, and reported reductions in local fish richness and abundance in the Sekong, Sesan and Srepok Basins (3S Basin) and altered flow seasonality in the 3S-Mekong-Tonle Sap system, with distinct variations in fish assemblages^16^. Yoshida et al.^12^ reviewed the impacts of mainstem hydropower dams on agriculture and fisheries resources, and found that annual fish catch has declined by 276,847 and 178,169 tonnes in Cambodia and Vietnam, respectively. Baird and Green^17^ and Green and Baird^18^ reviewed and discussed the impacts of dams on fish and ecosystems and provided options for future clean development. Soukhaphon et al.^19^ studied several Mekong hydropower dams and found that social and environmental impacts are often cumulative and occur over large scales. All of these studies highlight the significant and varied costs from hydropower development in the LMB and together suggest that further dam construction is not a sustainable development trajectory.

**Figure 1:**
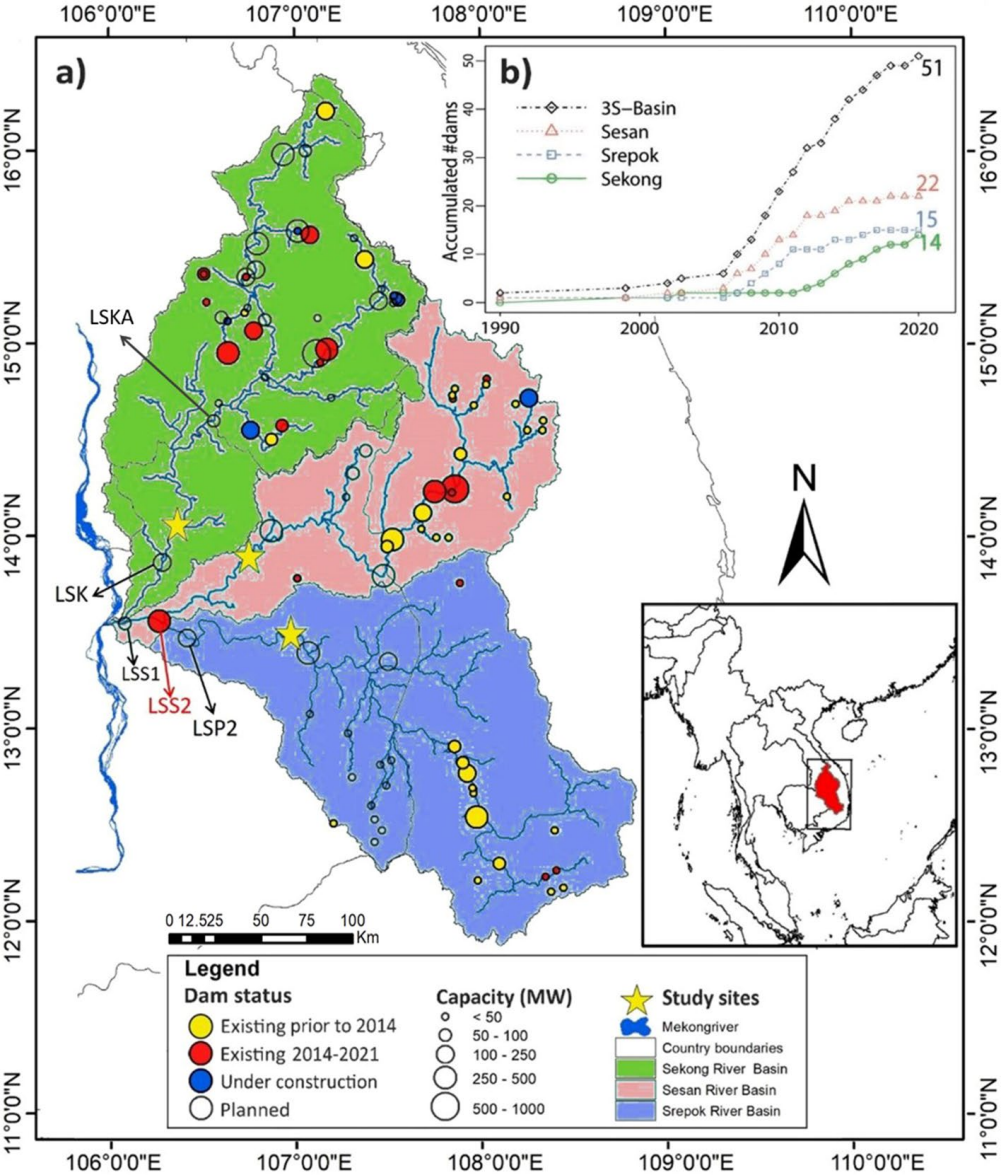
(**a**) Sampling sites and hydropower dams built in the 3S Basin and (**b**) number of dams from 1990 to 2021. Data source: Mekong Dam Monitor platform^13^. Lower Sesan 2 Dam (LSS2) began operations in 2018, and thus did not exist during the study period. (*LSK* Lower Sekong Dam, *LSKA* Lower Sekong A Dam, *LSS1* Lower Sesan 1 Dam, *LSP2* Lower Srepok 2 Dam). Map was created using ArcMap 10.4.1.

One of the hotspots for hydropower dam development is the Sekong, Sesan and Srepok Basins (3S Basin), which together form a major tributary to the Mekong. In 2007, the Sekong Basin had two dams, the Srepok Basin had two dams, and the Sesan Basin had six dams. In 2014, the number increased to six dams in the Sekong Basin, 13 dams in the Srepok Basin, and 19 dams in the Sesan Basin. Of those dams, six were major dams located on the mainstem river of the Sesan Basin, five were located on the mainstem of Srepok Basin, and one dam was located on the mainstem river of the Sekong Basin—although it was situated far upstream (Fig. 1a). By 2021, 51 dams were operational in the 3S Basin (Fig. 1b), with total generating capacity of 4684 MW. Five more dams are under construction and 79 are planned^13^. Despite the widespread impacts of hydropower dam development, limited research has quantified spatial and temporal changes in fish biodiversity with dam development.

The 3S Basin has vast ecological importance, with a seasonal flood-pulse that fuels exceptional aquatic biodiversity. Moreover, the 3S Basin is one of the main fish migration routes in the LMB, providing spawning and rearing habitats for some species^9,20^. Among the three Basins, the Srepok was historically utilized by 81 species of migratory fish, the Sekong by 64 species, and 54 species of migratory fish used the Sesan Basin^21,22^. The 3S-Mekong-Tonle Sap is a migratory corridor linking fish spawning and rearing habitats, including endangered species like the Critically Endangered Mekong Giant Catfish (*Pangasianodon gigas*) and Endangered Mekong River Catfish (*Pangasianodon hypophthalmus*). While the Mekong is home to at least 150 endemic aquatic fauna^23^, 103 of which are endemic fish species living in the LMB^24^, it also has approximately 20 non-native species^25^, which compete with native species for resources^3,24^.

Here, we assess spatial–temporal changes in fish biodiversity from hydropower dam development in the 3S Basin from 2007 to 2014. By using long-term monitoring data, we (1) quantify changes in fish biodiversity composition and indicator species in the 3S Basin, (2) investigate the temporal trends in fish biodiversity (richness, abundance and diversity indices of total, migratory and threatened fish species) and (3) quantify the impacts of dam development on fish biodiversity. We conclude with discussion about sustainable development futures for the 3S Basin.

## Results

### Overall fish biodiversity and spatial-seasonal changes

**Figure 2:**
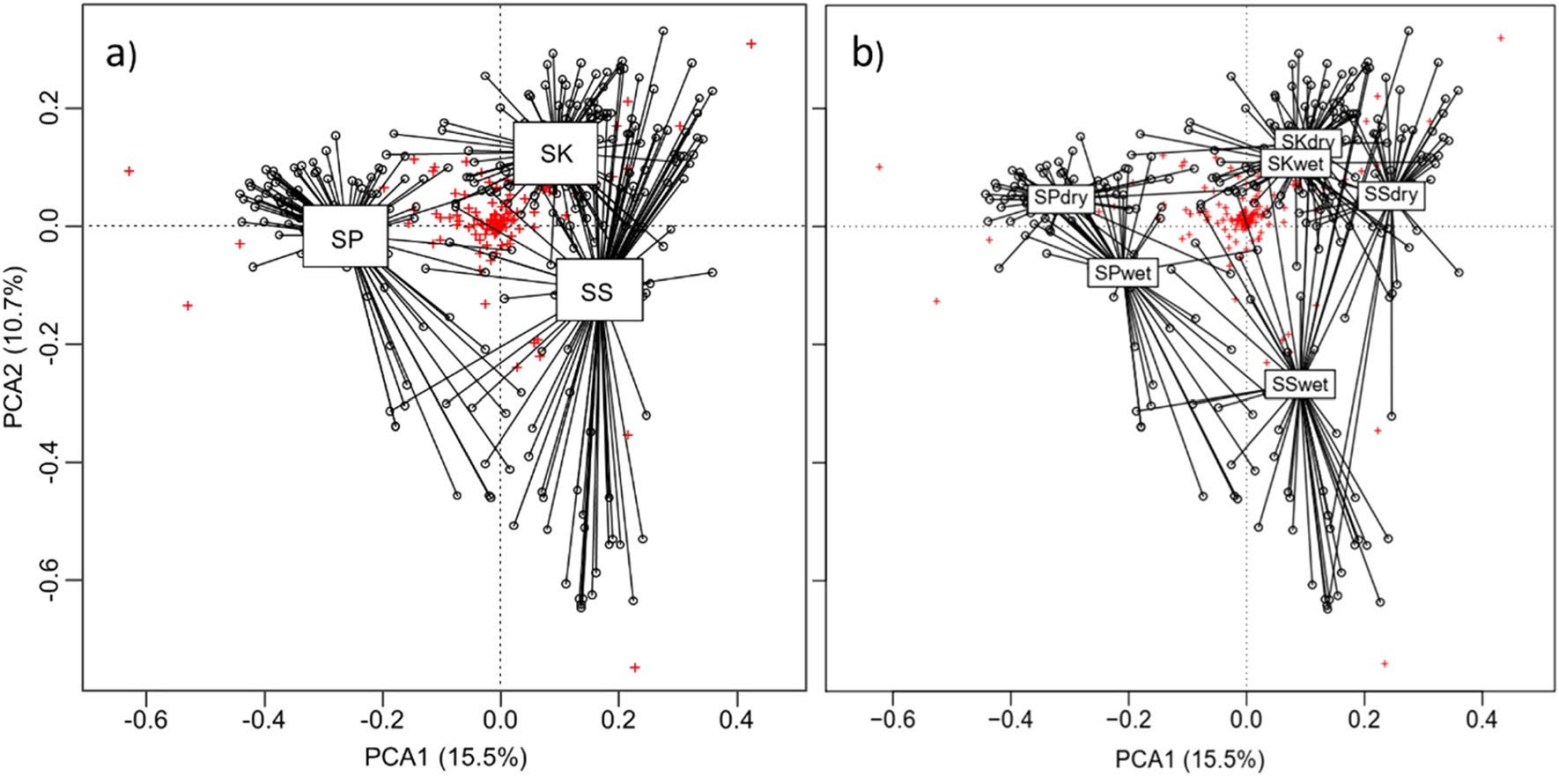
(**a**) Spatial and (**b**) seasonal-spatial fish community clusters by river basin based on Hellinger-transformed monthly fish community data (red crosses) collected from Sekong (SK), Srepok (SP), and Sesan (SS) Rivers, dry: dry season, wet: wet season. Figure was created using R statistical programing language version 4.0.1.

Our study area had 247 fish species belonging to 43 families and 14 orders. The most common order in the 3S Basin was Cypriniformes (52%, 129 species), followed by Siluriformes (24%, 60 species), and Perciformes (11%, 28 species). The remaining 11 orders made up less than 3% of the total species counts. PCA analysis demonstrated distinct fish communities among the 3S rivers (Fig. 2a) and changes in those fish communities between the wet and dry seasons in the Srepok and Sesan Basins (Fig. 2b).

We also characterized spatial-seasonal communities in each river by indicator species (Table 1). As whole, Sekong Basin had 19 indicator species, five of which were migratory and two were threatened species (*Mystus bocourti*—Vulnerable and *Pangasianodon hypophthalmus*—Endangered), whereas Srepok Basin had 10 indicator species, including 6 migratory and one Critically Endangered species (*Probarbus jullieni*). Sesan Basin had only one indicator species (*Acantopsis* sp.). Fish communities in Sekong Basin were relatively similar between seasons, as indicated by the PCA ordination map (Fig. 2b). In the Srepok Basin, there was one indicator species for the dry season and 6 indicator species in wet season. The Sesan Basin had 5 indicator species that characterized fish communities in the dry season and none in the wet season (Table 1).

**Table 1:** Indicator species list by river and season in the 3S Basin. Asterisks *P < 0.05, **P < 0.01, ***P < 0.001, *m* migratory, *VU* Vulnerable, *EN* Endangered, *CR* Critically Endangered, *nn* non-native, *end* endemic in Mekong. Values range from 0 to 1, and values near 0 indicate rare or sporadic abundance, while consistently abundant species at many sites near 1.

Species	Indicator value	Species	Indicator value
**Sekong—dry**		**Srepok—dry**	
* Barbodes rhombeus*	0.295*	* Pao baileyi*	0.267*^,*end*^
* Cynoglossus microlepis*	0.329**^,*m*^		
* Mystacoleucus chilopterus*	0.307*	**Srepok—wet**	
* Oreochromis niloticus*	0.285**^,*nn*^	* Barbodes binotatus*	0.334**
		* Channa gachua*	0.466**
**Sekong—wet**		* Clarias meladerma*	0.494**
* Channa lucius*	0.311*	* Clarias nieuhofii*	0.302*
* Lobocheilos gracilis*	0.309**	* Clarias gariepinus*	0.339**^,*nn*^
* Pseudecheneis sulcatoides*	0.244*	* Ompok bimaculatus*	0.570**
* Puntioplites waandersi*	0.267*		
* Tor laterivittatus*	0.283*	**Srepok—dry & wet**	
		* Cosmochilus harmandi*	0.897**^,*m*^
**Sekong—dry and wet**		* Helicophagus waandersii*	0.953**^,*m*^
* Arius venosus*	0.267*	* Hemibagrus wyckioides*	0.841**^,*m*^
* Barbonymus altus*	0.680**	* Labeo dyocheilus*	0.665**
* Barbonymus gonionotus*	0.850**	* Mystus atrifasciatus*	0.620**
* Cyclocheilichthys heteronema*	0.408**^,*m*^	* Mystus multiradiatus*	0.600**
* Discherodontus ashmeadi*	0.400**	* Mystus singaringan*	0.675**
* Heteropneustes kemratensis*	0.482**	* Pangasius conchophilus*	0.901**^,*m*^
* Labiobarbus lineatus*	0.393**^,*m*^	* Probarbus jullieni*	0.372*^,*m*,*CR*^
* Macrochirichthys macrochirus*	0.469**	* Pseudolais pleurotaenia*	0.750**^,*m*^
* Megalops cyprinoides*	0.284*		
* Misgurnus anguillicaudatus*	0.378**	**Sesan—dry**	
* Mystacoleucus atridorsalis*	0.267**	* Henicorhynchus lobatus*	0.782**^,*m*,*end*^
* Mystus bocourti*	0.695**^,*VU*^	* Lycothrissa crocodilus*	0.746**
* Osteochilus waandersii*	0.321*^,*m*^	* Puntioplites bulu*	0.681**^,*m*^
* Pangasianodon hypophthalmus*	0.336**^,*m*,*end EN*^	* Raiamas guttatus*	0.427**^,*m*^
* Pangasius kunyit*	0.362**^,*m*^	* Rasbora tornieri*	0.604**
* Puntioplites proctozysron*	0.725**		
* Rasbora daniconius*	0.267*	**Sesan—dry & wet**	
* Rasbora hobelmani*	0.568**	* Acantopsis* sp.	0.360**
* Rasbora myersi*	0.402**		

**Figure 3:**
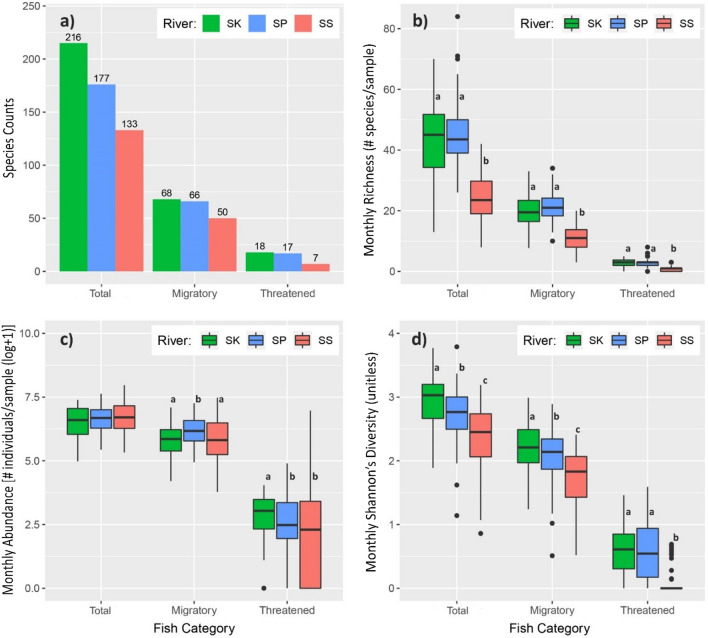
Fish biodiversity metrics for the Sekong (SK), Srepok (SP), and Sesan (SS) Rivers. (**a**) species counts, (**b**) monthly species richness, (**c**) monthly species abundance and (**d**) monthly diversity H. The common letters within each fish category boxplots indicated no significant difference at the P < 0.05 level. Figure was created using the R statistical programing language version 4.0.1.

The Sekong Basin had 216 fish species, 68 species were migratory and 18 were threatened species. The Srepok Basin had 177 species, with 66 migratory and 17 threatened fish species, and the Sesan Basin had 133 fish species, with 50 migratory and 7 threatened species (Fig. 3a). In the Sekong Basin, species belonged to 13 orders, from which Cypriniformes accounted for 51% (110 species), followed by Siluriformes (26%, 56 species), and Perciformes (11%, 23 species). In the Srepok Basin, fish belonged to 13 orders and were predominately represented by three orders: Cypriniformes (50%, 88 species), Siluriformes (26%, 45 species), and Perciformes (11%, 19 species). In the Sesan Basin, fish belonged to 8 orders. Cypriniformes (55%, 73 species), Siluriformes (29%, 38 species), and Perciformes (8%, 10 species) were the dominant orders. The full species list by genera, family, order, migratory guild, and ICUN red list category is provided in Supplemental Information Table S1.

We found significant differences in monthly fish diversity (referring to Shannon’s diversity index), species richness, abundance by fish category (all, migratory, and threatened species) between the river basins (Fig. 3b–d). The monthly richness of the three categories was not significantly different between the Sekong (42.2) and Srepok (45.2) Basins, but monthly richness was lower in the Sesan Basin (24.1) (Fig. 3b, Table 2). Monthly abundance was significantly higher in the Srepok Basin for migratory fish and in the Sekong Basin for threatened fish (Fig. 3c, Table 2). For the three fish categories, diversity were different among the basins, and diversity was highest in the Sekong Basin, followed by the Srepok Basin, and was lowest in the Sesan Basin (Fig. 3d).

**Table 2:** Descriptive statistics including mean, standard deviation, minimum and maximum monthly fish species richness (number of species/sample) and abundance (number of individuals/sample) in each monitoring site of each 3S river. Diversity: referring to Shannon’s diversity index (unitless). *Denotes significant difference between Basins for each corresponding metric based on a one-way ANOVA or a Kruskal–Wallis test. The common letters indicate no pair-wised significant difference, otherwise it has a significant difference.

Fish categories	Sekong		Srepok		Sesan	
Total fish	Mean ± SD	Min–Max	Mean ± SD	Min–Max	Mean ± SD	Min–Max
Richness*	42.2 ± 12.9^a^	13–70	45.2 ± 10.5^a^	20–84	24.1 ± 8.3^b^	8–44
Abundance (log + 1)	6.4 ± 0.7	4.9–7.4	6.7 ± 0.5	5.4–7.6	6.7 ± 0.6	5.3–8.3
Diversity*	2.9 ± 0.4^a^	1.9–3.8	2.6 ± 0.4^b^	1.1–3.8	2.4 ± 0.5^c^	0.9–3.2
Migratory fish						
Richness*	18.9 ± 5.6^a^	6–33	22.4 ± 4.8^a^	10–34	10.6 ± 4.2^b^	1–20
Abundance (log + 1)*	5.8 ± 0.7^a^	4.2–7.1	6.3 ± 0.6^b^	4.9–7.3	5.7 ± 0.9^a^	2.9–7.5
Diversity*	2.2 ± 0.4^a^	1.2–3.0	2.0 ± 0.4^b^	0.5–2.9	1.7 ± 0.5^c^	0.0–2.4
Threatened fish						
Richness*	2.7 ± 1.3^a^	0–6	2.8 ± 1.6^a^	0–8	0.9 ± 0.7^b^	0–3
Abundance (log + 1)*	3.0 ± 0.9^a^	0.0–4.5	2.4 ± 1.1^b^	0.0–5.1	2.1 ± 1.7^b^	0.0–6.9
Shannon’s diversity*	0.6 ± 0.4^a^	0.0–1.6	0.6 ± 0.5^a^	0.0–1.8	0.1 ± 0.2^b^	0.0–0.7

In the 3S Basin as a whole, 8 LMB-endemic fish species were observed, with seven, eight, and four species observed in the Sekong, Srepok and Sesan Basins, respectively. Four non-native species were detected throughout the 3S Basin. The Sekong and Srepok Basins each had three non-native species, while there were none in the Sesan Basin over the study period. Refer to Table S1 for detailed taxonomy.

### Temporal fish biodiversity trends and the impact of dam development

**Figure 4:**
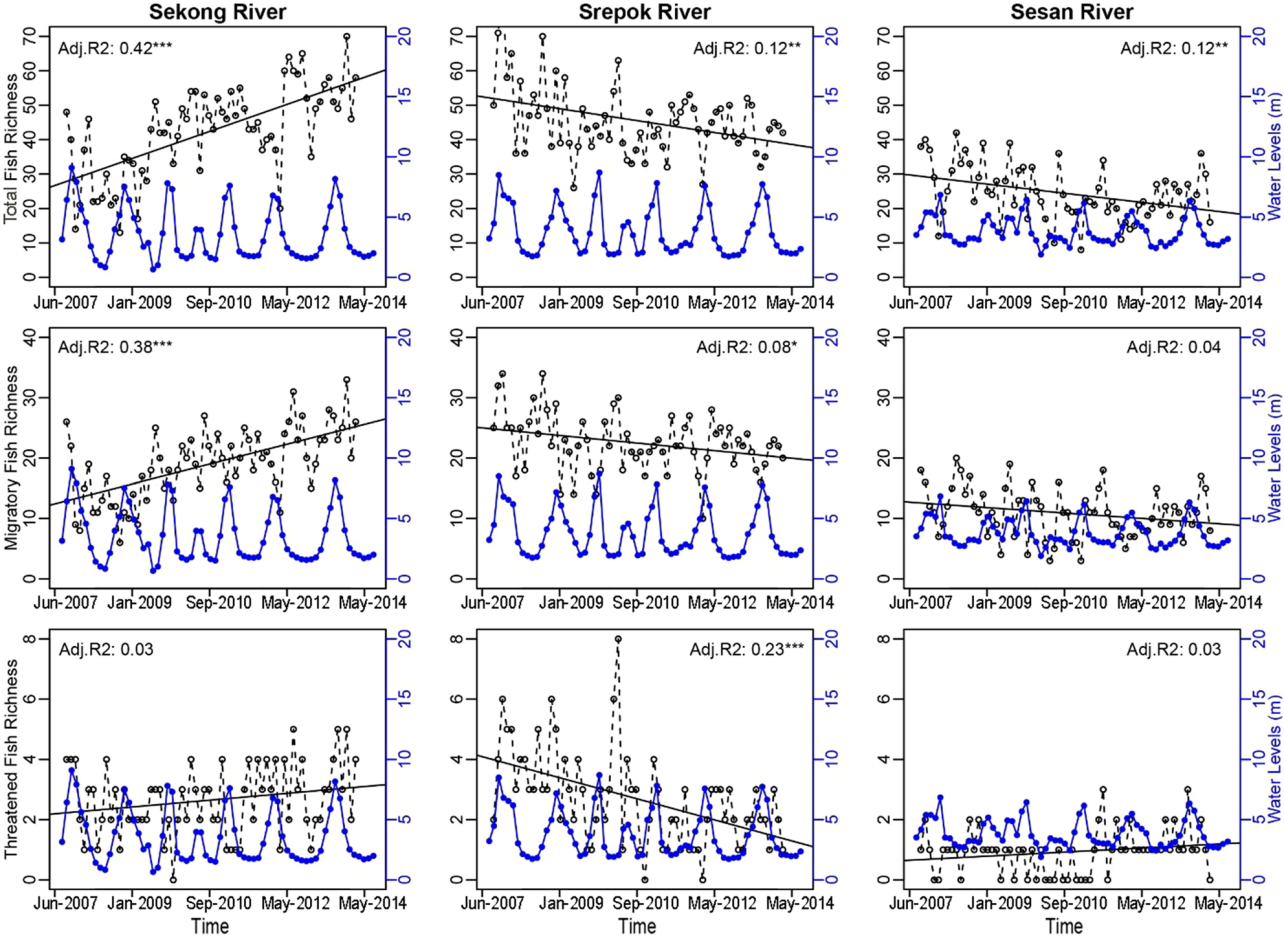
Monthly water levels from 2007 to 2014 (blue lines) and monthly fish biodiversity richness (number of species/sample, black dashed lines) in the Sekong, Srepok, and Sesan Basins. Asterisks *P < 0.05, **P < 0.01, ***P < 0.001. Figure was created using R statistical programing language version 4.0.1.

Overall, fish biodiversity metrics are increasing in the Sekong Basin and declining in the Srepok and Sesan Basins. Linear regression models indicated that fish biodiversity metrics (species richness, abundance, and diversity) in the Sekong Basin significantly increased from 2007 to 2014 for total fish and migratory fish. Threatened fish biodiversity metrics showed an upward trend for the Sekong Basin, but only diversity was significant (Fig. 4, Supplemental Information Fig. S1). In the Srepok Basin, fish richness, abundance, and diversity significantly declined from 2007 to 2014 for threatened species, while richness also significantly declined for total and migratory species. In the Sesan Basin, richness and diversity significantly declined for total species and only diversity significantly declined for migratory species (Fig. 4, Supplemental Information Fig. S1).

When the change in biodiversity metrics were significant, model performance (R^2^) ranged from 0.06 to 0.42 (Fig. 4, Supplemental Information Fig. S1). These significant but low R^2^ values mean that there was considerable noise in the data, or in other words, fish catch varied considerably among months and years and the trend for fish diversity was statistically significant. The highest R^2^ values indicated increasing total and migratory fish biodiversity in the Sekong Basin—where few dams were developed, and declining biodiversity for threatened fish in the Srepok Basin.

**Figure 5:**
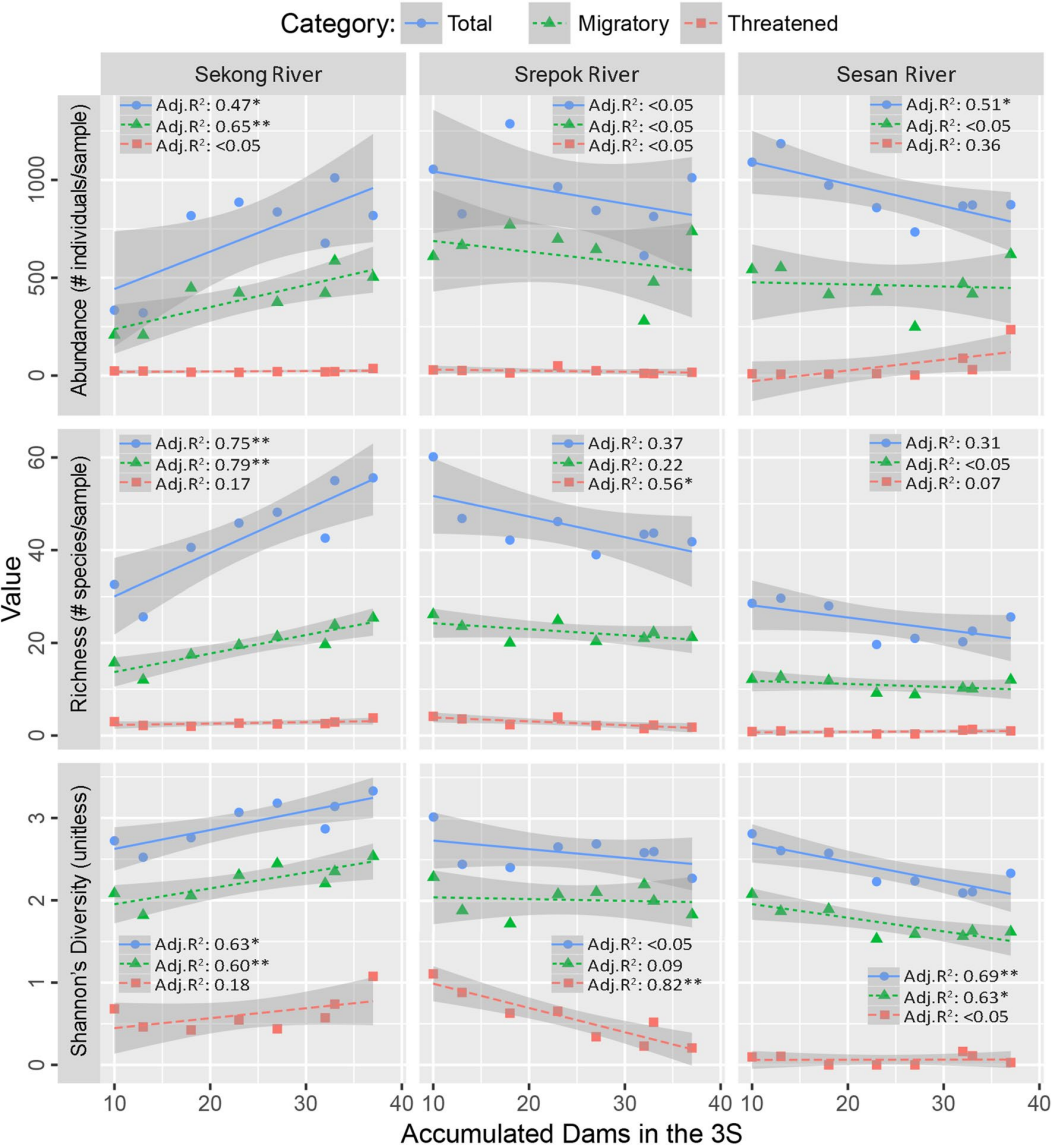
Linear regression models between fish biodiversity metrics in each river and accumulated dams in the 3S Basin. Adj.R2: adjusted coefficient of determination. Asterisks *P < 0.05, **P < 0.01.

Based on linear regression at an annual timestep, fish biodiversity metrics in the Sekong Basin increased significantly through time as dams were built in other tributaries of the 3S Basin, primarily the Sesan and Srepok Basins (Figs. 1, 5). A similar pattern was found when the data was partitioned into seasons, particularly in the dry season (Figs. S2, S3). Total fish richness in the Sekong Basin significantly increased from 33 species in 2007 to 56 species in 2014 with a model performance of R^2^ = 0.75 (*P* < 0.01). Abundance, richness, and diversity always significantly increased in the Sekong Basin for total species and migratory species, with R^2^ values ranging from 0.47 to 0.79. In contrast, biodiversity trends declined in the Srepok and Sesan Basins as more dams were built (Fig. 5). Monthly total fish richness in the Srepok and Sesan Basins decreased from 60 to 42 species and 29 to 25 species, respectively, from 2007 to 2014. In the Srepok Basin, richness and diversity of threatened fish species significantly decreased (R^2^ = 0.56 and R^2^ = 0.82, respectively); while in Sesan Basin, total fish abundance and diversity, and the migratory fish diversity significantly decreased (R^2^ = 0.51, R^2^ = 0.69 and R^2^ = 0.63, respectively) (Fig. 5).

## Discussion

Our results confirm that the 3S Basin is an important tributary system in the LMB, with impressive biodiversity. For example, the 247 species observed in the 3S Basin comprise 60% of the 411 freshwater fish species found in Cambodia^26^ and 22% of the 1148 fish species estimated for the entire Mekong Basin. Moreover, fish communities in the 3S Basin showed marked adaptation to each river basin, characterized by distinct spatial composition and indicator species (Fig. 2, Table 1). Two of the four non-native fish are indicator species, which suggest that the 3S now provides habitat for the non-native species and therefore could threaten native species by competing for resources^24^. Our findings also demonstrate that dams and fish biodiversity are inversely correlated. As the number of dams has increased in the Srepok and Sesan Basins, fish richness, abundance, and diversity have generally declined. On the other hand, fish richness, abundance, and diversity have increased through our study period in the relatively healthy and free-flowing rivers of the Sekong Basin (Fig. 4, Fig. S1).

Average total fish abundance over the study period was not significant different among the three rivers, but migratory fish abundance was significantly higher in the Srepok Basin, while threatened species abundance was significant higher in the Sekong Basin. However, monthly abundance significantly increased through time in the Sekong Basin for total and migratory fish (Fig. S1). Clear evidence of increased fish biodiversity in the Sekong Basin is illustrated by diversity, which significantly increased through time for all fish categories and which accounts for both species richness and their relative abundance^27^ (Fig. S1). The opposite results were found for the Srepok and Sesan Basins, specifically the significant decreasing diversity through time for threatened species, and for the total and migratory species, respectively (Fig. S1).

Our findings highlight the importance of the Sekong Basin as a free-flowing river corridor for fish migration and suggest that the Sekong Basin potentially compensates for environmental changes and habitat loss in the Sesan and Srepok Basins. The Sekong Basin had the highest number of total, migratory, and threatened fish species observed in the 3S Basin (Fig. 3a) and currently provides about 10,330 km of accessible habitat to migratory fish^21^. We counted more total and migratory fish species than past observations^22^. Sekong Basin fish communities included 28 indicator species across the study period, which means that these species were abundant and widespread in the Sekong Basin throughout the study period. The non-native species, Nile tilapia (*Oreochromis niloticus*), was an indicator species in the Sekong Basin because they were caught only in this basin and were abundant (Table S1). Although the detrimental impacts of this species in the Mekong Basin is not well understood, its presence could lead to the decline of native species^28^. This highlights the need to manage the 3S Basin, especially the Sekong Basin, to prevent future threats to native fish from non-native species. Furthermore, we found six migratory indicator species and two threatened indicator species (the Mekong endemic striped catfish *P. hypophthalmus* and the Vulnerable *M. bocourti*) in the Sekong Basin, highlighting the basin’s importance to migratory and threatened fish^29,30^.

As dams were built in the Sesan and Srepok Basins, fish diversity decreased. The Sesan is the most dammed river in the 3S Basin (Fig. 1b) and it supported the lowest fish biodiversity, confirming results from a previous study^9^. The Sesan Basin also had the lowest number of migratory, threatened, and indicator species, although in the past it was a migration route for at least 54 migratory species^22^. Five indicator species were recorded in the dry season, one of which is the Mekong endemic *Henicorhynchus lobatus*, indicating their long-term occurrence with high abundance only in the dry season. The Sesan Basin had no wet season indicator species. However, throughout the study period and regardless of season, the Sesan Basin had only one indicator taxon, *Acantopsis* sp., suggesting that fish species that were present in the beginning of the study period were replaced by different species by the end of the study period. The Srepok Basin was the second-most dammed river in the 3S Basin and supported more fish biodiversity and more indicator species than the Sesan Basin, although richness and diversity for threatened fish species have declined through time. The Srepok Basin historically supported the highest number of migratory species in the 3S Basin^9,21^. However, compared to past records by Baran et al.^22^, the number of migratory fish has declined by 15 species and total species count was reduced by 63 species by 2014.

Lower Sesan 2 Dam was completed in 2018 and disconnected the Srepok and Sesan Basins from the Mekong River (LSS2 in Fig. 1). That dam blocked 18,701 km of upstream habitat for fish migration. The presence of Lower Sesan 2 Dam alone underscores the importance of maintaining free-flowing rivers of the Sekong Basin. Our results are the first to demonstrate that fish abundance, richness, and diversity are increasing in the Sekong Basin as other tributaries are disconnected by dams. We have shown that dam construction in the 3S Basin during past decades have altered river ecosystems.

Our results mirror studies in other regions which have shown that dams are an overriding driver of native fish species loss^31^. For example, hydropower development has fragmented river systems in major Andean-Amazonian basins, especially for endemic and migratory species^32^. River fragmentation from future hydropower dam development is anticipated to severely impact fish movement in the Mekong, as well as Amazon, Niger, Congo, and Salween Basins^33^. The combination of climate change, drought intensification, and hydropower dam development is anticipated to harm fisheries and fish biodiversity^34^.

In the Mekong, hydropower is often promoted as a renewable, sustainable source of energy for economic development^14^. However, our study demonstrated that hydropower development causes fisheries and biodiversity loss. This finding contributes to existing research showing that dams degrade ecosystems by altering water flow and quality, blocking fish migration routes, and disturbing fish spawning and feeding grounds^9,35–38^. Free-flowing rivers and intact migration corridors are thus vital to sustaining future food webs, biodiversity, fisheries, and food security in the LMB^39,40^. Protecting migratory fish species is particularly important as they are the food source for threatened birds and freshwater mammals such as the Critically Endangered Irrawaddy Dolphin^41^. Migratory fish also make up about 60% of the catch in the LMB^42^ and are central to food security and nutrition because they provide 49–83% of animal protein for people living in the LMB^40^. Moreover, previous research has shown that dam construction indirectly leads to deforestation, displaced communities, increased socio-economic conflict, and deterioration of natural resource livelihoods like semi-subsistence farming, wild fishing and forestry^43–46^.

Given the negative impacts of hydropower dams on streamflow and water quality^21,47^, fish biodiversity and abundance, and ecosystem function, it is imperative to include realistic environmental impacts in future hydropower dam planning^21,48^. Our results quantified significant impacts of dams on fisheries in the Sesan and Srepok Basins, and possible compensatory benefits of a largely undammed Sekong Basin. The findings suggest that additional dams in the Sekong Basin, especially the proposed Lower Sekong (LSK) and the Lower Sekong A (LSKA) dams (Fig. 1a), which could be built in the lower mainstem river of Sekong Basin (LSK in Fig. 1a), would likely incur substantial environmental and social costs. Since the Srepok and Sesan Basins have already been dammed, maintaining connectivity in the Sekong Basin to support fish diversity and fisheries is needed and valuable. A largely free-flowing Sekong benefits local communities with diverse freshwater resources and wild fish capture^7,49,50^. Abundant and diverse fish can increase the catch and thus the enough daily protein intake for local people, and provide fisheries-based incomes. Moreover, the free-flowing Sekong can maintain relative natural-seasonal flows that upgrade river integrity and ecosystem services including regulation (e.g. water and erosion regulation, self-purification), provisioning (e.g. water supply and fisheries), and supporting services (e.g. soil formation, nutrient and water cycling). Seasonal agricultural activities can also benefit from the free-flowing Sekong because connected rivers ensure seasonal flow availability and support sediment transfer to healthy floodplains^51^, which all are necessary for agricultural production.

An alternative to future dams in the Sekong Basin is to promote other alternative renewable resources like solar energy or to re-operate existing dams to maximize hydropower generation without new dams. For example, solar energy combined with centralized power management and trading could enable Thailand, Laos, and Cambodia to meet future energy demand without new hydropower dams^42^. Similarly, operating multiple dams as a system, rather than operating dams independently, could optimize hydropower generation and reduce the need for new dams^52^. In the 3S Basin, 51 dams are already operational, and therefore hydropower re-operation, especially for large dams such as Lower Sesan 2, should be prioritized^53^. Alternative energy sources and optimizing hydropower generation of existing dams will be increasingly important as hydrologic intensification expected with climate change reduces hydropower generation from existing dams and reduces the benefits of proposed dams^54^.

## Methods

### Study area

The 3S Basin is one of the Mekong’s main tributaries and drains southern Laos, Vietnam’s central highland, and northeastern Cambodia, joining the Mekong mainstem in Stung Treng Province, Cambodia (Fig. 1a). The 3S Basin is important to Mekong River hydrology, in particular Tonle Sap’s flood pulse and seasonal reverse flow, because it contributes up to 25% of the Mekong’s annual discharge and sediment load^55^. Like the LMB, the hydrology of the 3S Basin has well-defined wet (May–October) and dry (November–April) seasons. More than 80% of annual rainfall occurs in the wet season. Across the 3S Basin, mean annual rainfall varies greatly from ~ 1500 mm in the lower reaches to ~ 2500 mm in the southern headwaters and > 3000 mm in the northern headwaters^56^.

### Data collection

Fish monitoring was conducted from June 2007 to May 2014 in the 3S Basin. Each of the three monitoring sites, that could extend a few kilometers in length, was located in each 3S river adjacent to the village where participating fishers were based (Fig. 1a). The three sampling sites remained relatively unchanged over the course of study. At each sampling site, three professional fishers, who are ‘typical’ medium‐scale full‐time fishers, were trained to record their daily catches by responsible researchers from the Inland Fisheries Research and Development Institute (IFReDI), Cambodia Fisheries Administration. After training, the fishers were tested to ensure their reliable ability in setting up fishing gears, identifying, measuring, recording and taking photos of fish species. For more detailed fisher training and testing, we refer to Ngor et al.^57^.

To reduce the effect of sampling effort among study sites, only fish catches from stationary gillnets (120 ± 50 m long, 2–3.5 m high, with mesh sizes of 3–12 cm) each day (12 ± 2 h per day) were used in this study. Fish were identified and counted using a fish species list (∼ 900 species) from the MRC Mekong Fish Database, FishBase, and other existing literature^1,58^. A fish photo book containing 272 common Mekong’s fishes including 20 non-native species with species code, local and scientific names was also provided to each participating fishers to aid fish identification and fish recording on the data sheet in the field^25^. The fish sampling procedures used in this study follows the MRC standard sampling procedures for Fish Abundance and Diversity Monitoring in the LMB described in Ngor et al.^57^.

### Data analysis

In this study, daily fish samples were averaged across all fishers for monitoring sites to daily mean samples, where were then aggregated into monthly fish community data. Monthly data were used to capture fish biodiversity response to seasonal hydrology and climate. Daily data added noise, but did not change monthly trends. Descriptive statistics including mean, standard deviation, minimum and maximum monthly fish species richness and abundance (total individuals catch of all species), and differences between the monitoring sites are provided in Table 2.

To assess spatial-seasonal changes in fish biodiversity in the 3S Basin, a principal component analysis (PCA) was performed using the *rda()* function of the “*vegan*” R package^59^. We used Hellinger-transformed data of the total monthly fish community using the *decostand()* function of the “*vegan*” R package^59^. This transformation reduced the impact of the highest fish abundance values^60^. PCA reduces dimensionality and summarizes the main features of a dataset. For example, data points may separate into clusters when samples are taken from different seasons or ecosystems or gradients of sites when a given biotic community is adapted to different conditions of the ecosystems^60^.

We also determined indicator fish species for each 3S river. An indicator fish species here is a species that occurs consistently through time, and more abundant compared to other species. Indicator species value ranges from 0 to 1. Indicator values near 0 indicate rare or sporadic abundance. When a species is consistently abundant in sites and is found at all sites of that group, and therefore has a large mean abundance within the group, its indicator value is 1. Smaller indicator values suggest that a species is not an indicator species; however, smaller indicator values do not necessarily preclude species from being indicators because the value also depends on the abundance of the species caught in each sample. We used the “*indicspecies*” R package^61^ to determine the indicator fish species and compute and test their indicator values against a *P* value of 0.05 based on a permutation test^60^. If any species has a significant indicator value, it is considered an indicator species. An ecosystem that supports a high number of indicator species can provide information on the characteristics and environmental conditions of the habitats and sites that they share^61^. A species was considered an indicator when its occurrence and mean abundance in the study site remained high throughout the study period, implying favorable ecological conditions at that site throughout the study period^60^. In this regard, a river that supports a number of fish indicator species may indicate that the river’s ecological conditions are favorable and stable over time. Otherwise, the fish species occurring in the river are merely visitors or do not occur consistently in the river due to hydrological or water quality fluctuations, habitat alteration, or river fragmentation.

Fish biodiversity was grouped into three fish categories: (1) total species, (2) migratory species and (3) threatened species. Each category differs in sensitivity to river conditions, river connectivity, and imminent risk to biodiversity. Migratory fishes undergo regular, predictable movements from one region to another within a larger system (www.fishbase.se), and threatened fish species are listed as Critically Endangered (CR), Endangered (EN) and Vulnerable (VU)^62^. The fish biodiversity metrics such as species richness, abundance and diversity (refereeing to Shannon’s diversity index) were used for all three fish categories for each 3S river. Species richness and species abundance refer to the number of different species and the total individuals of all species per sample or per given area, respectively. The Shannon’s diversity index is computed following the equation: diversity = -1Σ [p_*i*_ × ln (p_*i*_)]^27^. Here, p_*i*_ is the proportion of individuals of the *i*th species in a given community, and is computed as p_*i*_ = n/N, where *n* is the individuals of the *i*th species and *N* is the total number of individuals in the whole community. The metrics were compared to understand whether fish communities in the Sekong, Srepok, and Sesan Basins are distinct from one another. The comparison was based on one-way ANOVA using the *aov()* function in the “*stats*” R package, or Kruskal–Wallis tests using the *kruskal.test()* functions^63^. The former was applied when residuals of the Shapiro–Wilk test were normal; otherwise, the non-parametric test was used. When significant differences were detected, we performed Wilcoxon tests using the *wilcox.test()* function of the “*stats*” R package^63^ to evaluate the differences in fish biodiversity metrics among river basins.

To investigate the temporal trends in fish biodiversity, we employed linear models to regress the monthly biodiversity metrics of the three fish categories against time using the *lm()* function of the “*stats*” R package^63^. In total, there were 84 monthly data points of fish biodiversity. To quantify the impacts of dam development, each metric of the three fish categories from each river was averaged to yearly data and then regressed against the yearly number of hydropower dams that were constructed in the 3S Basin. This produced eight data points for the 2007–2014 sampling period. We used yearly data because data for the month that hydropower dams became operational in unavailable. All analyses were conducted using the R statistical programing language^63^ and values of *P* < 0.05 were considered significant. The map in this study was created using ArcMap version 10.4.1. All figures were generated using functions in the “*stats*” R package^63^ and the “*ggplot2”* R package^64^.

### Ethical statements

All field and measurement protocols were approved by the Inland Fisheries Research and Development Institute (IFReDI), Cambodia Fisheries Administration, and all methods were carried out in accordance with relevant guidelines and regulations of IFReDI. Fish catch and handling after all scientific measurement and records was granted to local fisher for consumption and sale by Fisheries Capture Committee, and the need for ethics approval is deemed unnecessary according to the regulations of IFReDI.

## Supplementary Information

Supplementary Information 1.

Supplementary Information 2.

Supplementary Information 3.

Supplementary Information 4.

## Data Availability

Data used for this study is available at: https://www.hydroshare.org/resource/bc362bf77750428d9b722942b8dc160d/.
