# Supplementary material for: Fish biodiversity declines with dam development in the Lower Mekong Basin
Source: Sci Rep. 2023 May 26;13:8571. doi: 10.1038/s41598-023-35665-9 (PMC10220217; doi:10.1038/s41598-023-35665-9)
Supplement: Supplementary file 2 — Supplementary Information 2. [file 41598_2023_35665_MOESM2_ESM.docx]

# Supplemental Information Figure S1

**Title:** Fish biodiversity declines with dam development in the Lower Mekong Basin

**Authors:** Ratha Sor ^1,2,3,^*, Peng Bun Ngor ^3,4^, Sovan Lek^5^, Kimsan Chann^6^, Romduol Khoeun^6^, Sudeep Chandra^7^, Zeb S. Hogan^7^, Sarah E. Null^1^

^1^ Department of Watershed Sciences, Utah State University, Logan, UT 84322, USA

^2^ Graduate School, National University of Cheasim Kamchaymear, No. 157, Preah Norodom Blvd, Khan Chamkarmon, Phnom Penh 12300, sCambodia

^3^ Wonders of the Mekong Project, c/o IFReDI, Fisheries Administration, No. 186, Preah Norodom Blvd., Khan Chamkar Morn, Phnom Penh 12300, Cambodia

^4^ Faculty of Fisheries, Royal University of Agriculture, Sangkat Dongkor, Khan Dongkor, P.O. Box 2696, Phnom Penh 120501, Cambodia

^5^ Université de Toulouse, Laboratoire Evolution & Diversité Biologique, UMR 5174, CNRS - Université Paul Sabatier, 118 route de Narbonne, 31062 Toulouse cédex 4 – France

^6^ Department of Water and Environmental Engineering, Institute of Technology of Cambodia, Russian Boulevard, Phnom Penh 12000, Cambodia

^7^ Global Water Center & Department of Biology, University of Nevada, 1664 N. Virginia Street, Reno, NV 89557, USA

* Correspondence: [sorsim.ratha@gmail.com](mailto:sorsim.ratha@gmail.com)


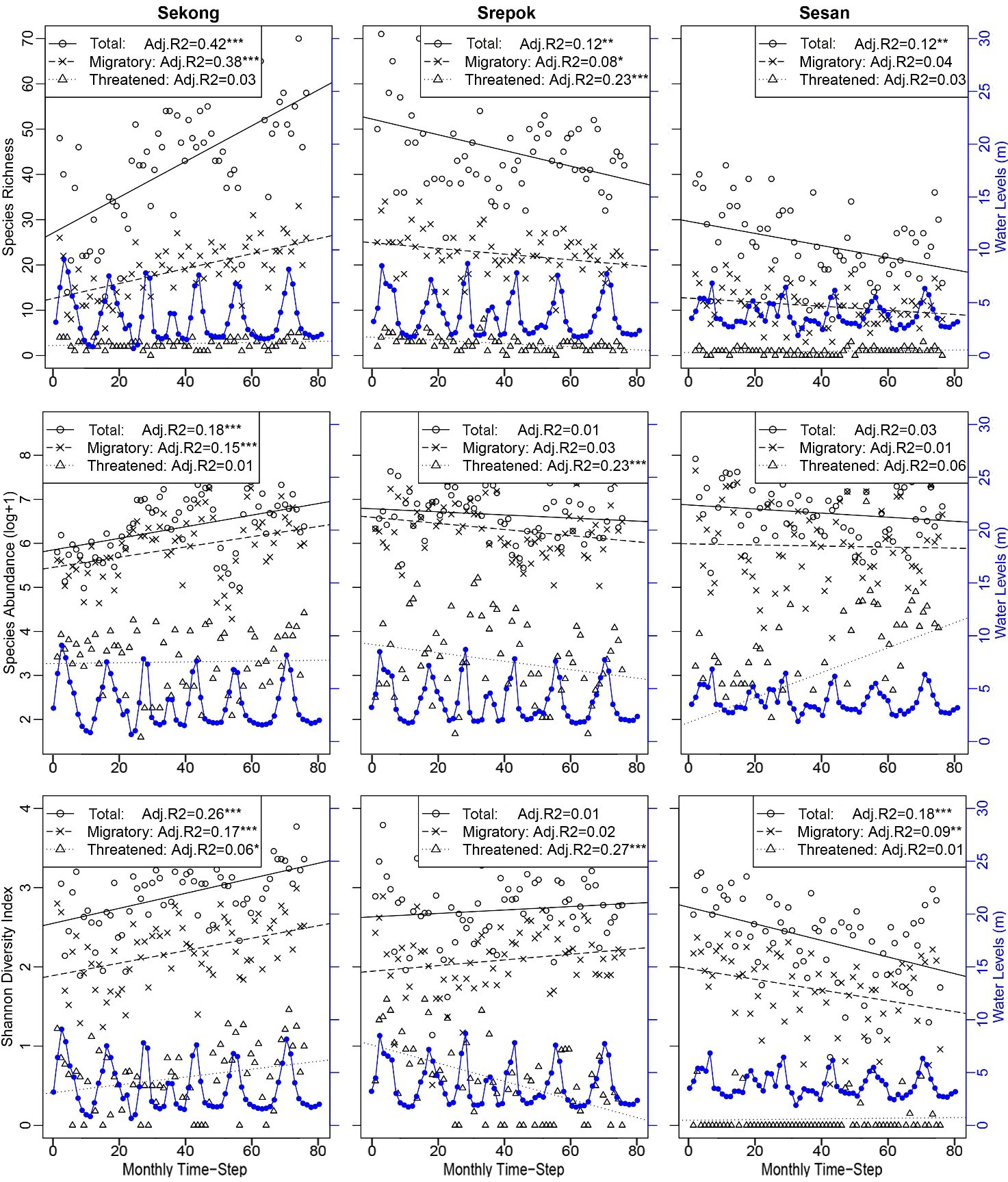


**Figure S1** Linear regression results indicating increasing fish biodiversity richness, abundance and Shannon diversity index through time (monthly time-step) in the Sekong River Basin and decreasing fish biodiversity richness, abundance and Shannon diversity index through time in the Srepok and Sesan River Basins. Blue lines are seasonal water levels from 2007 to 2014. Asterisks *: P<0.05, **: P<0.01, ***: P<0.001.
