# Supplementary material for: Fish biodiversity declines with dam development in the Lower Mekong Basin
Source: Sci Rep. 2023 May 26;13:8571. doi: 10.1038/s41598-023-35665-9 (PMC10220217; doi:10.1038/s41598-023-35665-9)
Supplement: Supplementary file 4 — Supplementary Information 4. [file 41598_2023_35665_MOESM4_ESM.docx]

# Supplemental Information Figure S3

**Title:** Fish biodiversity declines with dam development in the Lower Mekong Basin

**Authors:** Ratha Sor ^1,2,3,^*, Peng Bun Ngor ^3,4^, Sovan Lek^5^, Kimsan Chann^6^, Romduol Khoeun^6^, Sudeep Chandra^7^, Zeb S. Hogan^7^, Sarah E. Null^1^

^1^ Department of Watershed Sciences, Utah State University, Logan, UT 84322, USA

^2^ Graduate School, National University of Cheasim Kamchaymear, No. 157, Preah Norodom Blvd, Khan Chamkarmon, Phnom Penh 12300, Cambodia

^3^ Wonders of the Mekong Project, c/o IFReDI, Fisheries Administration, No. 186, Preah Norodom Blvd., Khan Chamkar Morn, Phnom Penh 12300, Cambodia

^4^ Faculty of Fisheries, Royal University of Agriculture, Sangkat Dongkor, Khan Dongkor, P.O. Box 2696, Phnom Penh 120501, Cambodia

^5^ Université de Toulouse, Laboratoire Evolution & Diversité Biologique, UMR 5174, CNRS - Université Paul Sabatier, 118 route de Narbonne, 31062 Toulouse cédex 4 – France

^6^ Department of Water and Environmental Engineering, Institute of Technology of Cambodia, Russian Boulevard, Phnom Penh 12000, Cambodia

^7^ Global Water Center & Department of Biology, University of Nevada, 1664 N. Virginia Street, Reno, NV 89557, USA

* Correspondence: [sorsim.ratha@gmail.com](mailto:sorsim.ratha@gmail.com)


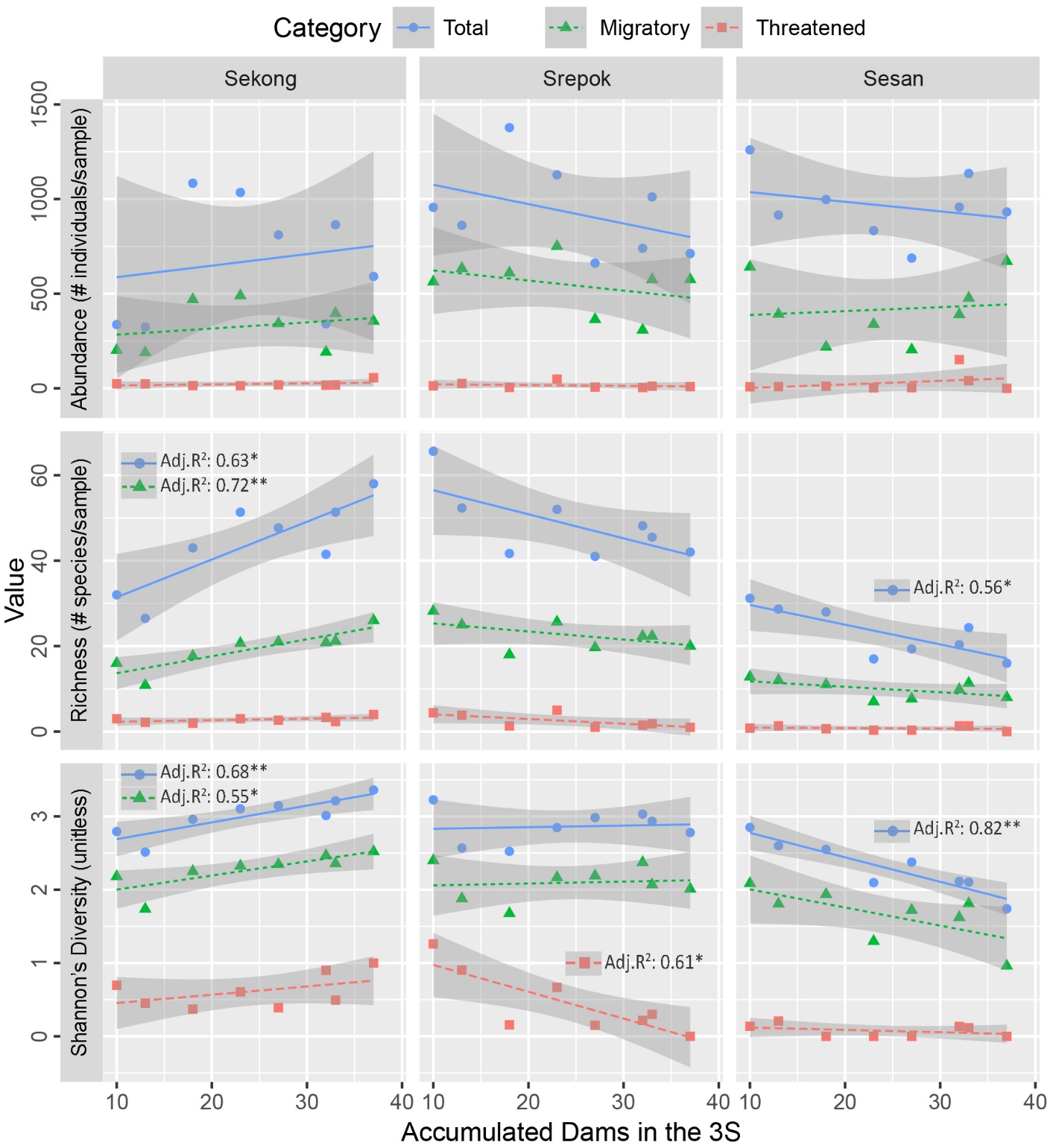


**Figure S2** Linear regression models between fish biodiversity metrics in wet season in each river and accumulated dams in the 3S Basin. Adj.R2: adjusted coefficient of determination. Asterisks *: P<0.05, **: P<0.01.
